# COVID-19: How to Reduce Some Environmental and Social Impacts?

**DOI:** 10.5334/aogh.3104

**Published:** 2020-11-02

**Authors:** Christian Voirol, Julia Sader, Marie-Claude Audétat

**Affiliations:** 1 Haute Ecole Arc Santé, HES-SO University of Applied Sciences and Arts Western Switzerland, Espace de l’Europe, Neuchâtel, CH; 2 University of Montreal, Faculty of Medicine, Department of family medicine and emergency, Montréal, CA; 3 University of Montreal, Faculty of Arts and Sciences, Department of psychology, Montréal, CA; 4 Unité de Développement et de Recherche en Éducation Médicale (UDREM) and iEH2 – Institut Éthique Histoire Humanités, Faculté de Médecine, University of Geneva, Rue Michel-Servet, Genève, CH; 5 Unité de développement et de recherche en éducation médicale (UDREM) and Unité des internistes généralistes et pédiatres (UIGP), Faculté de Médecine, University of Geneva, Rue Michel-Servet, Genève, CH

## Abstract

The proposed viewpoint seems important at this time of the COVID-19 pandemic. Indeed, the COVID-19 prevention strategies implemented in the population are based on medical paradigms that generate extremely deleterious social and environmental impacts. Using the occupational health and safety perspective – taking care of myself with recyclables PPE (Personal Protective Equipment) – in place of the medical perspective – taking care of others with disposable PPE – can help to influence and support this important public health reflection.

The COVID-19 pandemic caught our society off guard. As expected, the voice of physicians and health care professionals prevailed at the beginning of the pandemic. After that, lawyers, followed by economists who came and put into perspective the health discourse. Finally, ethicists and philosophers came to debate the underlying threats to our freedom. In this deafening cacophony, perhaps it is time to restore the concern and perspective of environmentalists? Indeed, before the pandemic, there was a vivid discussion around removing plastic straws from the food industry (e.g. fast-food restaurants). Today, we use and throw away billions of surgical masks, and it seems to go unnoticed. A recent survey has just documented the presence of surgical masks on the Mediterranean seabed [1]. SARS-CoV-2 is probably here to stay. Therefore, we should revisit the policies and strategies put in place by the public health, by strategies that will be functional in the long run.

## A public health policy that produces mountains of waste

Numerous studies reinforced how face masks have helped slow down the spread of COVID-19 [2]. Recent studies [3,4] have explored how wearing a surgical mask is a protective measure. For example, one study suggests that mandatory mask wearing policies are linked to the reduction in face-touching behavior, which may help to prevent contact transmission of COVID-19 in the general population [5]. Only if everyone wears a form of mask (surgical or otherwise) can the protection of the community be heightened [4]. The use of surgical masks stems from a hospital paradigm that aims to first and foremost protect the patient. However, face mask use remains controversial, due to the lack of hindsight and limited data, as well as how the general population understand and use these masks [4]. Nevertheless, wearing a surgical mask, a face shield and a physical distance of at least one meter reduces the risk of contamination of the Personal Protective Equipment (PPE) wearer by about 35% (10.2% + 14.3% + 10.6% = 35.1%) [6]. If necessary, N95/FFP3 masks can be used by the caregiver for a more efficient barrier to the virus [7]. Whilst a significant difference between a surgical mask and a N95/FFP3 mask does not seem to have been demonstrated for the prevention of COVID-19 [8,9], so-called “general public” masks are proven to be less effective [10]. Once again, only through an adherence of the community can the effectiveness of such masks be heightened [11]. This being said, this preventive strategy is based on the use and destruction of huge quantities of surgical or general public masks. If over the next 18 to 24 months, six to eight billion people use one or more surgical masks a day, we will face a new environmental crisis linked to this production of waste that has become unmanageable.

However, there are recyclable half face masks (Half Facepiece Elastomeric Respirators) to which it is possible to connect specific filters for aerosols (P2) [12]. These masks filter out between 95% and 99% of particles [13]. The cost of these masks is approximately $25 each. Depending on the country’s resources, access to more sustainable masks might be challenging. The high price will unfortunately not make it accessible to most, and this issue is one that has to be further explored [14]. These recyclable face masks are also more comfortable to wear and almost as easy to use as disposable masks [15], as well as easy to maintain. The use of such masks produces much less waste, as the filters require to be changed after several hundred hours of use. To date, these filters remain relatively expensive because they are made for industrial use and protection from exposure to toxic gases or vapours. In the current context, this would essentially be a low-soiling urban use. We would hope that manufacturers of such masks are able to develop new filters, less expensive and specifically adapted for COVID-19 use. In fact, recently, MIT researchers published a study presenting a prototype recyclable mask specifically designed for COVID-19 [16]. In doing so, it would be possible to massively reduce the waste generated by the strategies currently advocated by public health agencies around the world. If necessary, subsidizing and making these Half Facepiece Elastomeric Respirators available to the population could be integrated into policies to preserve the environment and the climate.

## Reversing the “protection” paradigm

There is, therefore, an opportunity to limit the environmental disaster that will result from the release of billions of surgical masks into the environment. But this alternative requires a major paradigm shift: Moving from a hospital centred paradigm which focused on protecting the patient, to a personal protection paradigm which is used in the Occupational Health and Safety (OHS) field [17,18]. Like N95/FFP3 masks, the air exhaled through the valve by the wearer is not filtered. In other words, these masks do not protect others; they protect the wearer. Thus, collective measures may be adopted upstream, in the end, in the OHS paradigm.

By defining a COVID-19 prevention policy in the public domain, which is based on the hospital rule of patient protection, we have reversed the logic used in OHS. For example, in public transport, by using surgical masks, we essentially protect other users. But in return, we expect to be protected. If this is not the case, then the injustice felt is expressed through an ethical discourse on collective responsibility and respect for others. And in return, those questioned express unfounded obligations and an infringement of their individual freedoms. This public health approach, based on collective responsibility and the protection of others, is probably unsuitable and too complex to implement in Western societies, which are accustomed to much more individualistic approaches. In industrial hygiene and particularly in high-risk professions (firefighters, military, linemen, etc.), we know how complex it is to put one’s own safety in the hands of one’s colleagues at work [19,20]. It requires a high level of trust. We have neither the time nor the ability to develop this trust at the collective level. And the constraint and obligation to wear a mask leads to socially unsatisfactory reactions [21]. Given these facts, what remains is to promote a strategy of individual protection, using reusable PPE, whereby each individual takes responsibility for his or her own protection.

## Interdisciplinarity to manage the complexity of our new collective reality

Since the beginning of this pandemic, health care providers have been working daily to refine their clinical scripts on COVID 19, i.e. to develop and organise a knowledge network integrating diagnostic, investigative, and therapeutic aspects [22]. Similarly, our society as a whole has to develop knowledge and coping strategies and will have to reinvent a way of living in the presence of this virus. If in the crisis, this reflection was essentially based on mono-views based on disciplinary skills, it is essential now, given that it will last, to develop an interdisciplinary and systemic reflection. Thus, instead of trying to determine whether it is doctors, lawyers, economists, philosophers, ethicists or environmentalists who are right, it is time to set up interdisciplinary reflections. Such reflections are likely to foster the emergence of efficient and sustainable responses. In this sense, suggesting the reopening of the economy and basing prevention policy on a strategy that massively produces special waste and unbearable infringements on our fundamental freedoms is an example not to be followed. The challenges at hand are clearly complex and require systemic and interdisciplinary thinking to find relevant, sustainable solutions [23,24].
